# Harbingers of Hope: Scientists and the Pursuit of World Peace

**DOI:** 10.32872/cpe.13197

**Published:** 2023-12-22

**Authors:** Seithikurippu R. Pandi-Perumal, Willem A. C. M. van de Put, Andreas Maercker, Stevan E. Hobfoll, Velayudhan Mohan Kumar, Corrado Barbui, Arehally Marappa Mahalaksmi, Saravana Babu Chidambaram, Per Olof Lundmark, Tual Sawn Khai, Lukoye Atwoli, Vitalii Poberezhets, Ramasamy Rajesh Kumar, Derebe Madoro, Hernán Andrés Marín Agudelo, Samuel Ratnajeevan Herbert Hoole, Luísa Teixeira-Santos, Paulo Pereira, Konda Mani Saravanan, Anton Vrdoljak, Miguel Meira e Cruz, Chellamuthu Ramasubramanian, Alvin Kuowei Tay, Janne Grønli, Marit Sijbrandij, Sudhakar Sivasubramaniam, Meera Narasimhan, Eta Ngole Mbong, Markus Jansson-Fröjmark, Bjørn Bjorvatn, Joop T. V. M. de Jong, Mario H. Braakman, Maurice Eisenbruch, Darío Acuña-Castroviejo, Koos van der Velden, Gregory M. Brown, Markku Partinen, Alexander C. McFarlane, Michael Berk

**Affiliations:** 1 Saveetha Medical College and Hospitals, Saveetha Institute of Medical and Technical Sciences, Saveetha University, Chennai, India; 2 Division of Research and Development, Lovely Professional University, Phagwara, Punjab, India; 3 Section of International Health Policy, Institute for Tropical Medicine Antwerp, Antwerp, Belgium; 4 Institute of International Humanitarian Affairs (IIHA), Fordham University, Bronx, NY, USA; 5 Psychopathology and Clinical Intervention, University of Zurich, Zurich, Switzerland; 6 STAR Consultants – STress, Anxiety and Resilience, Salt Lake City, UT, USA; 7 Kerala Chapter, National Academy of Medical Sciences (India), New Delhi, India; 8 WHO Collaborating Centre for Research and Training in Mental Health and Service Evaluation, Department of Neuroscience, Biomedicine and Movement Sciences, Section of Psychiatry, University of Verona, Verona, Italy; 9 Department of Pharmacology, JSS College of Pharmacy, JSS Academy of Higher Education and Research, Mysuru, India; 10 Department of Optometry, Radiography and Lightning Design, University of South-Eastern Norway, Kongsberg, Norway; 11 National Centre for Optics, Vision and Eye Care, Kongsberg, Norway; 12 School of Graduate Studies, Lingnan University, Hong Kong SAR, China; 13 Medical College East Africa, Brain and Mind Institute, The Ag​a Khan University, Nairobi, Kenya; 14 Department of Propedeutics of Internal Medicine, National Pirogov Memorial Medical University, Vinnytsya, Ukraine; 15 All India Institute of Training and Education (AIITE), New Delhi, India; 16 Department of Psychiatry, College of Medicine and Health Sciences, Dilla University, Dilla, Ethiopia; 17 Behavioral Sleep Medicine Institute, Neumocenter, Research Department, Medellín, Colombia; 18 Baldaeus Theological College, Adukubar, Konesapuri, Trincomalee, Sri Lanka; 19 Center for Health Technology and Services Research, Porto, Portugal; 20 Nursing School of Coimbra, Coimbra, Portugal; 21 Environmental Management Laboratory, Mykolas Romeris University, Vilnius, Lithuania; 22 Department of Biotechnology, Bharath Institute of Higher Education & Research, Chennai, India; 23 Faculty of Civil Engineering, Architecture and Geodesy, University of Mostar, Mostar, Bosnia and Herzegovina; 24 Centro Cardiovascular da Universidade de Lisboa, Lisbon School of Medicine, Lisbon, Portugal; 25 Division of Community Psychiatry, M. S. Chellamuthu Trust and Research Foundation, Madurai, India; 26 Discipline of Psychiatry and Mental Health, School of Clinical Medicine, University of New South Wales, Sydney, NSW, Australia; 27 Department of Biological and Medical Psychology, University of Bergen, Bergen, Norway; 28 Department of Clinical, Neuro- and Developmental Psychology, WHO Collaborating Center for Research and Dissemination of Psychological Interventions, Amsterdam Public Health Research Institute, Vrije Universiteit Amsterdam, Amsterdam, The Netherlands; 29 Department of Biotechnology, Manonmaniam Sundaranar University, Tirunelveli, Tamil Nadu, India; 30 Department of Neuropsychiatry and Behavioral Science, School of Medicine, University of South Carolina, Columbia, SC, USA; 31 MOMENTUM Integrated Health Resilience (MIHR), IMA World Health, Goma, North Kivu, Democratic Republic of Congo; 32 Centre for Psychiatry Research, Department of Clinical Neuroscience, Karolinska Institute, Stockholm, Sweden; 33 Stockholm Health Care Services, Region of Stockholm, Stockholm, Sweden; 34 Department of Global Public Health and Primary Care, University of Bergen, Bergen, Norway; 35 Norwegian Competence Center for Sleep Disorders, Haukeland University Hospital, Bergen, Norway; 36 Cultural Psychiatry and Global Mental Health, Amsterdam UMC, Amsterdam, The Netherlands; 37 Boston University School of Medicine, Boston, MA, USA; 38 Transcultural Forensic Psychiatry, Tilburg Law School, Department of Criminal Law, Tilburg University, Tilburg, The Netherlands; 39 Department of Psychiatry, School of Clinical Sciences at Monash Health, Monash University, Clayton, Victoria, Australia; 40 Centro de Investigación Biomédica, Departamento de Fisiología, Facultad de Medicina, Instituto de Biotecnología, Universidad de Granada, Granada, Spain; 41 Public Health, Department of Primary and Community Care, Radboud University Medical Center, Nijmegen, The Netherlands; 42 Centre for Addiction and Mental Health, Department of Psychiatry, University of Toronto, Toronto, Canada; 43 Sleep Medicine, Helsinki Sleep Clinic, Helsinki, Finland; 44 Department of Clinical Neurosciences, Clinicum, University of Helsinki, Helsinki, Finland; 45 Psychiatry, Faculty of Health and Medical Sciences, The University of Adelaide, Adelaide, South Australia, Australia; 46 The Institute for Mental and Physical Health and Clinical Translation (IMPACT), School of Medicine, Deakin University, Barwon Health, Geelong, Australia

**Keywords:** SDGs, UN, UNHCR, COVID-19, global health diplomacy, Hamas, Israel, mental health, psychiatry, Middle East, military invasion, Palestine, peace, scientist, sustainable development goals, war

## Abstract

The ongoing wars in many regions—such as the conflict between Israel and Hamas—as well as the effects of war on communities, social services, and mental health are covered in this special editorial. This article emphasizes the need for international efforts to promote peace, offer humanitarian aid, and address the mental health challenges faced by individuals and communities affected by war and violence.

*“Now I am become Death, the destroyer of worlds”*

- The Bhagavad Gita

The world has recently endured COVID-19, followed by the Russian invasion of Ukraine, the worldwide outrage about unprovoked invasions and subsequent deaths of civilians, and, currently, the deadly war between Israel and Hamas in Gaza appears to have no end in sight. In the context of a long duration of bad blood between Palestinians and Israelis, Hamas attacked Israel in early October, an invasion which was evidenced by videos of murder, rape, torture, and kidnapping of civilian hostages. At the time of writing, while Hamas continues its rocket attacks on Israeli cities, Israel has responded with a ground invasion and massive bomb strikes, activities that have resulted in incremental deaths among civilians, a particular heart-wrenching tragedy in Gaza, with its very high population ratio of children and youth. The massive death and destruction have raised alarms among all nations. What is badly needed is humanitarian assistance in Gaza, where the population lacks food, water, and fuel. Israel, in the meanwhile, has lost the hostages in Gaza but also many of its young men and young women who are now waging war on five fronts.

Fear reigns on both sides. More civilians are being killed every day, injured, displaced, bereaved, traumatized, and deprived of home and livelihood. Fear of the outbreak of a regional conflict has spread beyond Gaza and Israel, and large-scale public demonstrations are taking place around the world, especially on Western university campuses where Palestinians are viewed as the underdogs in an unequal war.

We, as scientists and clinicians, have the means, whenever possible, of relieving anxiety and emotional distress. Hence, we feel the need to make our voices heard in the midst of this crisis. There are turning points in history that require the dissemination of good sense.

Armed conflicts significantly undermine the economic vitality of conflict-affected nations (Seleznova et al., 2023) and severely harm their social, physical, and human capital, both during and after the conflict is over. If international organizations are not given the opportunity and support to take urgent action, a humanitarian disaster will take place in Gaza. Amidst overwhelming despair and a general feeling of helplessness, we want to use our experience in researching the consequences of war and violence on mental health to lay out the facts regarding the impact of war on civilization.

The effects of violence, from Israel and Gaza to Afghanistan, Cambodia, the Democratic Republic of Congo, Iraq, Libya, Syria, Yemen, Myanmar, Nepal, Rwanda, Sri Lanka, Syria, South Sudan, Sudan, and Ukraine, have all been well studied (Familiar et al., 2021; Kienzler & Sapkota, 2020; Razjouyan et al., 2022; Sá et al., 2022). History has supplied unambiguous evidence of the lasting harm of warfare (Hyseni Duraku et al., 2023; Leshem et al., 2023; Saw et al., 2023; Thomas et al., 2023). International wars, civil wars, proxy battles, conflicts, invasions, and insurgencies all end badly. They are all accompanied and followed by disruptions in the delivery of basic social services, especially access to healthcare, which has led to epidemics and spikes in infection and diseases, critical battle-related injuries, and chronic disability (Blais et al., 2023), acute malnutrition, acute and chronic mental health conditions, and horrific deaths (Sher, 2023). Wars always result in widespread suffering, enduring stress, trauma, loss, and population displacement, which can reverberate and scar the well-being of future generations. This, in turn, leads to the continuation of violence across generations (Betancourt, 2015; Castro-Vale et al., 2019; Dashorst et al., 2019). In longstanding conflicts, past injustices are used as rationales for future retribution and aggression. Human beings tend to ruminate over past grievances and, thus, view retaliation as justified. Continued violence rips at the social fabric of society, and healing is difficult, but can be achieved (Kapshuk & Deitch, 2023). It was achieved, against all odds, in Northern Ireland (Uluğ et al., 2023).

Living in war-torn countries has been associated with physical handicaps, and mental and psychological anguish (due to exposure to death). Complaints such as post-traumatic stress disorder (PTSD), anxiety, depression, sleeplessness, nightmares (Birhan et al., 2023; Pavlova et al., 2023; Rogowska & Pavlova, 2023), alcohol and substance abuse (Dissanayake et al., 2023), suicidal thoughts, tendencies, and attempted suicides (Blais et al., 2023; Sher, 2023) sexual and non-sexual violence (Hladik et al., 2023), and psychosomatic disorders all have been reported. These will have long-lasting effects on affected individuals. Both aggressors and victims face immense mental challenges: war trauma, violations of human rights, social exclusion, discrimination, spiralling rates of family violence, poverty, and loss of social support.

Women and children are disproportionately impacted because they are unable to flee from danger due to their socioeconomic dependence on men (Bendavid et al., 2021). Parental loss and family disruption negatively affect children throughout their adult lives, partly because the memory of the terrors of war impairs the parenting abilities of survivors (Ugurlu et al., 2016). Children grow up with attachment difficulties and personality problems and remain, throughout life, at high risk of suicide. Soldiers who serve in combat are increasingly reported as suffering from the often-catastrophic effects of injuries, medical problems such as chronic pulmonary disorders, as well as post-traumatic stress (Jordans et al., 2009).

Decades of rehabilitation and rebuilding work are always required to aid in the recovery of individuals impacted by war as well as in the restoration of communities and the rebuilding of means of subsistence. Many losses, not only of life but also of cultural traditions and meaningful religious symbols and structures, are irreversible. When examining the effects of war on mental health and well-being, the results are invariably catastrophic whether for winners or losers, combatants or civilians.

One of the most visible impacts of living in war-torn countries has been physical disability. Wars disrupt the supply chain of food and potable water, contributing to malnutrition, gastrointestinal and respiratory problems, as well as an increase in community infectious diseases. Wars disrupt youth development and education (Gómez-Restrepo et al., 2023), leaving lasting transgenerational impacts on individuals and society. A notable concern is the mental health of first, second, and third generations of survivors.

Refugees are highly susceptible to trauma. As noted by the United Nations High Commissioner for Refugees (UNHCR), the main reasons for fleeing one’s country are related to war, the threat to survival, and the violation of human rights. These situations undermine mental health. Fleeing brings with it the need to embrace a survival journey that involves abandoning one’s identity and self-worth, leaving one’s family and friends, subjecting oneself to dangerous, illegal crossings, and often needing to rely on unreliable human smugglers. Parental loss and family disruption adversely affect migrants for life (Raturi & Cebotari, 2023). The inhumane conditions in which people seeking asylum are forced to live while awaiting international protection are disastrous for mental health. We know that human beings are resilient (Purgato et al., 2020) but eventually, a limit to resilience is reached.

In the present world situation, we need to prepare for the worst. Specific physical, psychological, and mental health promotional help will be needed. Psycho-educational, psychological, and other integrated health services will be required, as suggested by the recently released World Mental Health Report (World Health Organization, Noncommunicable Diseases and Mental Health Cluster, 2005). Actions are needed to scale up interventions that are effective and sustainable in promoting mental health and preventing the development of mental disorders (Tol et al., 2023). As proposed by WHO, in civil societies, basic training in mental health and mental health first aid for people in civil societies should be a concern of all governments. Thousands of persons will be displaced and dispersed in host countries (Teixeira-Santos et al., 2023). There will be resource constraints and cost escalations. All sides in a conflict must make concessions, and this is difficult when there are wide differences in social, religious, and cultural norms, traditions, and values. A large influx of internally displaced people fleeing from violence at home is increasingly putting strains on the healthcare systems, other social services, and economies of countries that welcome refugees (Somasundaram et al., 2023). There are compromises that civilians, host governments, and communities need to make so that refugees can integrate into the host society and contribute to their new country’s economic growth. If such an agenda fails, it will put pressure on the existing fabric of our global system and this usually leads to political unrest down the road.

Disinformation campaigns spread misinformation, disinformation, mal-information, tendentious information, and alternative facts on both sides of a war conflict. As a result, even the well-intentioned fall prey to incorrect certainties, which they convey through their social networks. This results in unnecessary polarization and pitches neighbour against neighbour, destroying social networks that would be needed to re-establish the backbone of societies. The authors of this paper, as scientists and medical professionals, believe that our efforts are best focused on (i) averting conflicts among ourselves as a global community of scholars, scientists, and practitioners (not an easy feat); (ii) analyzing the effects of war; (iii) assisting in the creation of relief efforts, (iv) developing and studying the beneficial effects of new mental health promotion and prevention strategies; and (v) planning for mental health resources for now and for the (ideally quick) return of peace.

This special editorial has briefly highlighted some of the ramifications of war. When a conflict occurs in any part of the world, it triggers ripple effects that render us all vulnerable to fear. To combat fear, we unite as scientists to voice our opposition to war in general, as contrasted to protesting the rightness of one cause versus another.

As scientists, we strongly encourage international leadership and diplomacy among statesmen as a path to enduring peace (Pandi-Perumal et al., 2022). Scientific evidence has the power to improve the world's health, equality, justice, resilience, and prosperity for all. Negotiations and compromise among partners in dialogue lead to far better outcomes than mutual killings. International helping organizations and impartial, well-respected international leaders are crucial agents in advancing peace initiatives and giving civilians in war-torn regions, refugees, and internally displaced persons the much-needed assistance they require. As Nelson Mandela pointed out:

“*Negotiation and discussion are the greatest weapons we have for promoting peace and development.”*

The United Nations (UN) is the driving force behind the Sustainable Development Goals (“Addressing Sustainable Development through Economic Empowerment,” 2019); this is an intergovernmental set of objectives that advocates for 17 goals and 169 targets, covering a wide range of sustainable development issues and measured through 230 individual indicators that are inextricably linked to peace and stability (United Nations, n.d.). The SDGs are imperiled by conflicts that derail the process and prevent the aspiration of achieving critical milestones; this affects not only the countries directly involved in the conflict, but all nations.

Peace means better quality of life and better mental health. There is abundant scientific evidence that vulnerable populations, the public, planetary health and safety, global security, and the global economy must be protected in these very dangerous times. As scientists, we are mindful of the challenging, intricate, multifaceted, and malignant consequences of wars. Apart from its impact on human health, it also hurts biodiversity, accelerates climate disasters, and intensifies social inequalities, inequities, and injustices. Let’s form a multi-stakeholder partnership involving scientists, policymakers, legislators, and regulators to facilitate a sustainable future for our planet Earth.

## Data Availability

Data sharing not applicable to this article as no datasets were generated or analyzed during the current study.
